# The effects of short-term home-based exercise training via video call in older adults with physically inactive and sedentary life

**DOI:** 10.1007/s11845-025-03981-w

**Published:** 2025-06-18

**Authors:** Fazıl Kulakli, İlker İlhanli, İlker Fatih Sari

**Affiliations:** 1 Department of Physical Medicine and Rehabilitation, Faculty of Medicine, Giresun University, 28200 Giresun, Turkey; 2 Department of Physical Medicine and Rehabilitation, Faculty of Medicine, Ondokuz Mayıs University, Samsun, Turkey

**Keywords:** Exercise, Physically inactive, Sedentary Life, Video Calling

## Abstract

**Objectives:**

Investigating the physical and mental effects of short-term exercise program and motivation with video communication methods such as telephone and internet applications on physically inactive and sedentary older adult's daily lives.

**Methods:**

Thirty participants were reached by video calling method. Participants were evaluated twice before and after the program. A 6-min walk test (6MWT), Barthel index for activities of daily living, Tinetti performance oriented mobility assessment (Tinetti POMA) and Geriatric depression scale (GDS) were applied. The standard daily exercise program was applied to all participants for 1 week. Exercise programs were created as endurance, strengthening, stretching and balance exercises.

**Results:**

All participants (16 female and 14 male) completed the program. In terms of 6MWT, Tinetti POMA and GDS, a statistically significant improvement was found after the program compared to the pre-program (*p* < 0.001, *p* < 0.001 and *P* < 0.001, respectively).

**Conclusions:**

It is possible and effective to organize exercise programs and to follow these with distance video calling applications in physically inactive and sedentary older adults.

## Introduction

Physical inactivity is defined by the World Health Organization as not meeting the recommended levels of physical activity for each age group and is the fourth leading cause of death worldwide [1]. Sedentary behavior, however, is not the same as physical inactivity. It refers to any activity where the body is in a resting position, such as lying down, reclining, or sitting, and involves an energy expenditure of less than 1.5 MET [1, 2]. During the COVID-19 pandemic, individuals aged 65 and older were more likely to experience severe illness and higher death rates, leading many countries to impose quarantines on this age group. As these individuals could not leave their homes, telehealth and telerehabilitation became essential for accessing healthcare services [3].

In this study, we aimed to investigate the physical and mental effects of short-term exercise program and motivation on older adult's daily lives with video communication methods such as telephone and internet applications in physically inactive and sedentary older adults.

## Materials and Methods

The inclusion and exclusion criteria were applied to evaluate individuals over the age of 65 who applied to the Giresun Training and Research Hospital and the Ondokuz Mayıs University Faculty of Medicine Physical Medicine and Rehabilitation outpatient clinic.

### Inclusion criteria

- Medical records should be available in the last 6 months.
- Patients who are physically inactive and engage in less than 150 min of moderate-intensity aerobic exercise per week [4].
- Individuals with a Godin Leisure-Time Exercise Questionnaire score of < 14, indicating sedentary behavior [5].
- Must agree to participate in the program.
- Video call should be possible with phone or internet applications.

### Exclusion criteria

- Older adults who cannot tolerate tests or exercise program: Those with uncontrolled systemic disease (Pulmonary, cardiac, renal, oncological etc.), neuromusculoskeletal system disease, cognitive dysfunction, visual or auditory disorder.
- Those whose blood pressure cannot be measured by themselves or other residents.
- Older adults who are not able to communicate with video call via phone or internet.
- Those who do not have consent for the study.

GPower 3.1 software was used to calculate the required sample size. As a result of the power analysis using the data of a previously published study, we calculated an effect size 0.614 [6]. Based on a power of 80% and a 2-tailed a of 0.05, we calculated that the sample size required as 23 patients. Thirty older adults ≥ 65 years old were included in the study. The present study was approved by the Research and Ethics Committee of Clinical Studies linked to Giresun Training and Research Hospital, under the 16.01.2023/02 approval number written consent of the volunteers was obtained. The study was conducted in accordance with the principles of the Declaration of Helsinki.

The exercise program was introduced to the participants through a face-to-face session at the hospital. Demographic and clinical data including age, gender, education level, marital status, number of household members, height, weight, and known medical conditions were recorded. Participants were subsequently contacted via video calls, through which all assessments were conducted. Additionally, the exercise protocol was reviewed again during the first video call. To enhance patient adherence, maintain motivation, and address any potential questions, participants were contacted through brief video calls over the course of one week. All participants were evaluated twice before and after the program. There may be a performance difference between the face-to-face evaluation at the hospital and the evaluation to be made with a video call at home. Therefore, the evaluation before and after the exercise program was carried out with video call. For the evaluation, a 6-min walk test (6MWT), Barthel index for activities of daily living, Tinetti performance oriented mobility assessment (Tinetti POMA) and Geriatric depression scale (GDS) were applied. During these sessions, a physician specialized in Physical Medicine and Rehabilitation guided a model who acted as a patient. Through this approach, participants received both auditory and visual instruction regarding the assessment procedures. This method was implemented to enhance clarity, ensure consistency, and support accurate self-performance during the evaluations.

### Six-minute walk test [7]

The 6-min walk test (6MWT) was employed to assess functional capacity, a key indicator of morbidity and mortality in older adults as well as in cardiac, pulmonary, and neuromuscular conditions. The test measures the maximum distance a participant can walk on a flat, hard surface. Individuals with contraindications noted in their medical history or those ineligible for emergency intervention were excluded. Participants rested for at least 15 min prior to testing, and blood pressure and heart rate were recorded before and after the test. The test was discontinued if participants experienced chest pain, severe dyspnea, leg cramps, unsteadiness, pallor, or excessive sweating.

### Barthel index for activities of daily living [8]

The Barthel Index is one of the most widely used tools to assess activities of daily living (ADLs). It evaluates functions such as continence, feeding, hygiene, dressing, transfers, toileting, mobility, stair climbing, and bathing. Each item is scored based on the level of independence, with higher scores indicating greater functional autonomy.

### Tinetti performance oriented mobility assessment [9]

The Tinetti Performance-Oriented Mobility Assessment (POMA) evaluates balance and gait, primarily to assess fall risk in older adults. It includes 13 balance items (scored 0–2) and 9 gait items (scored 0–1), with a maximum total score of 35. Lower scores indicate a higher risk of falling. The test is widely used due to its clinical reliability and sensitivity to changes over time.

### Geriatric Depression Scale

The Geriatric Depression Scale (GDS) was developed by Yesavage et al. in 1983 to screen for depression in older adults [10]. The 15-item short form, validated by Burke et al. in 1991 [11], is a quick and practical tool. Scores above five suggest possible depression and warrant further clinical evaluation. Its applicability to patients with dementia is a notable advantage. The scale's reliability and validity in Turkish have also been demonstrated [12].

### Exercise program

The standard daily exercise program was applied to all participants for 1 week in total, one set in the morning. The exercise programs consisted of three main sections: warming up, working and cooling.

The purpose of warming up was to prepare the body for exercise. Movements were rhythmic and natural, smoothly transitioning from one movement to another. Circular movements were performed for the wrist, ankle, shoulders, neck and waist. Also, respiratory exercise was performed.

Puffy Lip Breathing: In a sitting position, after breathing through the nose for a few seconds while the mouth is closed, the lip contracts and breathes slowly in 4–6 s as if it whistles.

Diaphragmatic Respiratory Exercises: One hand is placed on the abdomen while the other hand is placed on the chest in a sitting or supine position. By slowly breathing through the nose, the hand on the abdomen is allowed to move upwards. While exhaling, it is pressed lightly on the abdomen and the lips are contracted and exhaled through the mouth.

Deep Breathing Exercise: In a sitting position, hands are placed on the lower ribs. Breathe deeply through the nose and feel the chest expanded. The intake air is held for 3–5 s. Then, puffy lips are exhaled slowly until it is felt that all the inhaled air is released. These were repeated 10 times. Total warming period was determined as 20 min.

Exercise programs were created from four types of exercises: Endurance, strengthening, stretching and balance exercises.

It was asked to walk at a pace that will not be out of breath and talk while walking for 20 min on the route determined for 6MWT as endurance exercise.

Bottles filled with water were used for strengthening exercises. Weight training was performed for the lower and upper extremities by asking to lift the weights in the plastic bag. The study was started with small weights (1.5 kg in the first three days) and increased from the third day (2.5 to 5 kg on the 4-7th days) and performed in 2 sets with 8–15 repetitions. The weight was lifted for 3 s and returned to the first position in 3 s. A few seconds rest period was given between repetitions.

For balance exercise, subjects were asked to stand as long as they can stand on one leg while standing and then change feet. As another balance exercise, they were asked to do a heel walk as much as they could. In addition, the subjects were asked to sit on the chair and do the lifting movement without using their hands.

Stretching exercises were shown separately for the back, upper and lower extremities, and were asked to be done with 3–5 repetitions of each muscle for 20–30 s. Stretching exercises were used for cooling, too.

The entire exercise period is limited to 60 min.

## Statistical Analysis

Statistical analysis was performed using the SPSS version 22.0 software (SPSS Inc., Chicago,

IL, USA). Analysis of skewness and kurtosis was used for testing the assumption of normality and values between −1,5 and + 1,5 was considered as providing the assumption of normality. Median (minimum–maximum) or mean ± standard deviation was given according to the assumption of normality. Mann–Whitney U test was used for the data not providing the assumption of normality where t-test was used for the opposite. Comparison of pre- and post-program was done with paired t test. Chi-square tests for categorical data and Pearson correlation tests were used as well. P value < 0.05 was considered statistically significant.

## Results

The study included 30 participants (16 women, 14 men), all of whom completed the program and assessments. The mean age was 71.3 years, and the average BMI was 30.08 kg/m^2^. Most were married and lived with one other person. Nearly half were high school graduates. Median walking distance was 1000 m (50–3000) in women and 1500 m (100–5000) in men. Gender-based comparisons are shown in Table 1.

**Table 1:** Demographic variables of the subjects

Gender		Female *N* = 16 (53.3%)	Male *N* = 14 (46.7%)	*P* Value
Age, years		69 (65–84)	70.50 (67–82)	0.275
BMI, kg/m^2^		33.40 (22.90–40.80)	27.90 (27.50–35.50)	0.019*
Marital status	Married	11 (68.8%)	12 (85.7%)	0.399
	Widow	5 (31.3%)	2 (14.3%)	
Education	Literate	3 (18.8%)	0	0.001*
	Elementary school	8 (50%)	0	
	High school	3 (18.8%)	11(78.6%)	
	University	2 (12.5%)	3 (21.4%)	
Number of households	1	1 (6.3%)	1 (7.1%)	0.541
	2	8 (50%)	10 (71.4%)	
	3	3(18.8%)	1(7.1%)	
	≥ 4	4 (25%)	2 (14.3%)	
Walking distance, meter		1000 (50–3000)	1500 (100–5000)	0.580

Median (minimum–maximum) values, subject numbers (frequencies)

^*^Significance level *p* < 0,05, BMI: Body Mass Index

Pre- and post-exercise data are presented in Table 2. Following the exercise program, participants showed significant improvements in mobility, balance, and mood. The 6MWT distance increased from 453.67 ± 171.12 to 474.88 ± 166.09 m (*p* < 0.001). Tinetti balance, gait, and total scores also improved significantly (all *p* ≤ 0.001), indicating better stability and reduced fall risk. The Barthel Index remained unchanged. GDS scores decreased from 8.56 ± 3.46 to 5.46 ± 4.03 (*p* < 0.001), suggesting reduced depressive symptoms.

**Table 2:** Comparison of the tests before and after exercise program

	Before exercise program (mean ± SD)	After exercise program (mean ± SD)	P value
6MWT, meter	453.67 ± 171.12	474.88 ± 166.09	< 0.001*
Tinetti POMA balance	19.73 ± 4.18	21.26 ± 4.20	< 0.001*
Tinetti POMA walking	8.06 ± 0.94	8.40 ± 0.81	0.001*
Tinetti POMA total	27.80 ± 4.97	29.66 ± 4.90	< 0,001*
Barthel	No difference		Not computed
GDS	8.56 ± 3.46	5.46 ± 4.03	< 0.001*

^*^Significance level *p* < 0.05, *SD*: standard deviation, *6MWT*: 6-min walk test (meter), Tinetti POMA balance: Tinetti performance oriented mobility assessment balance score, Tinetti POMA walking: Tinetti performance oriented mobility assessment walking score, Tinetti POMA total: Tinetti performance oriented mobility assessment total score, Barthel: Barthel index for activities of daily living score, GDS: Geriatric depression scale score

As presented in Table 3, age was significantly negatively correlated with 6MWT-B (r = –0.620, *p* < 0.01), POMAB-B (r = –0.759, p < 0.01), POMAW-B (r = –0.657, *p* < 0.01), and POMAT-B (r = –0.762, *p* < 0.01). Additionally, age showed a significant positive correlation with GDS-B (r = 0.694, *p* < 0.01). BMI was significantly negatively correlated with 6MWT-B (r = –0.395, *p* < 0.01) and 6MWT-A (r = –0.397, p < 0.01). Strong positive correlations were also observed between pre and post program scores of functional tests. Specifically, 6MWT-B was highly correlated with 6MWT-A (r = 0.993, *p* < 0.01), POMAB-B with POMAB-A (r = 0.977, *p* < 0.01), POMAW-B with POMAW-A (r = 0.862, *p* < 0.01), and POMAT-B with POMAT-A (r = 0.972, *p* < 0.01). Other correlation coefficients are also presented in Table 3.

**Table 3:** Correlation of variables

	BMI	6MWTB	6MWTA	POMABB	POMABA	POMAWB	POMAWA	POMATB	POMATA	GDSB	GDSA	BARTHEL
Age	‒.100	‒.620^**^	‒.632^**^	‒.759^**^	‒.737^**^	‒.657^**^	‒.723^**^	‒.762^**^	‒.750^**^	.694^**^	.724^**^	‒.691^**^
BMI		‒.395^*^	‒.397^*^	‒.281	‒.268	‒.348	‒.246	‒.302	‒.270	.154	‒.057	.035
6MWT-B			.993^**^	.803^**^	.795^**^	.849^**^	.843^**^	.836^**^	.820^**^	‒.642^**^	‒.540^**^	.586^**^
6MWT-A				.792^**^	.784^**^	.853^**^	.843^**^	.827^**^	.811^**^	‒.664^**^	‒.575^**^	.578^**^
POMAB-B					.977^**^	.807^**^	.822^**^	.994^**^	.973^**^	‒.782^**^	‒.673^**^	.600^**^
POMAB-A						.786^**^	.845^**^	.970^**^	.996^**^	‒.777^**^	‒.683^**^	.613^**^
POMAW-B							.862^**^	.868^**^	.816^**^	‒.677^**^	‒.606^**^	.622^**^
POMAW-A								.855^**^	.889^**^	‒.744^**^	‒.689^**^	.722^**^
POMAT-B									.972^**^	‒.786^**^	‒.681^**^	.622^**^
POMAT-A										‒.788^**^	‒.699^**^	.645^**^
GDS-B											.912^**^	‒.565^**^
GDS-A												‒.577^**^

^*^p < 0.05; ** *p* < 0.01 significance level

*BMI* Body Mass Index 6MWT-B: 6-min walk test before 6MWT-A 6-min walk test after, POMAB-B: Tinetti performance oriented mobility assessment balance before POMAB-A Tinetti performance oriented mobility assessment balance after POMAW-B: Tinetti performance oriented mobility assessment walking before POMAW-A: Tinetti performance oriented mobility assessment walking after POMAT-B: Tinetti performance oriented mobility assessment total before POMAT-A: Tinetti performance oriented mobility assessment total after GDS-B: Geriatric depression scale score before GDS-A: Geriatric depression scale score after

## Discussion

Elderly individuals are at risk of being physically inactive. Being physically active has positive effects on the risk of cardiovascular diseases, diabetes mellitus (DM), obesity, and cancer. People who are moderately or highly physically active have a lower risk of these mortality-increasing factors compared to inactive individuals [13]. According to the World Health Organization, physical inactivity ranks as the fourth leading cause of death globally, accounting for 6% of deaths. Studies show that physical activity can reduce the risk of diseases that impair quality of life, decrease functionality, and lead to morbidity and mortality in elderly individuals [14]. International guidelines recommend at least 150 min of moderate-intensity physical activity per week [4].

Globally, 31% of people do not meet the recommended levels of physical activity. Among individuals over the age of 55, the prevalence of physical inactivity in Europe ranges between 5 and 29% [14]. Although patients are advised on how much exercise they should do during outpatient visits, it is observed that they do not engage in sufficient levels of exercise, and even if they do, it cannot be adequately monitored to ensure it is performed correctly. This situation has highlighted the need to develop new methods for tracking physical activity. During the pandemic, telehealth systems came to the forefront, particularly due to the quarantine of elderly individuals and the challenges people faced in accessing healthcare services [15, 16]. Based on this, we aimed to investigate the short-term effectiveness of home-based exercises monitored via phone or internet in physically inactive, sedentary elderly patients, regardless of whether these conditions were influenced by the pandemic or not.

As we expected at the end of the present study, we determined the positive contributions of the exercise program. There was a significant improvement in 6MWT and Tinetti POMA results of the participants. This result showed us that although the exercise program duration is as short as one week, it will contribute positively to the performance, aerobic capacity, balance and walking of the subjects. In a previous study [6], it was stated that a 10-week exercise program combined with home-based walking program was effective on 6MWT and quality of life of older adults. Difference from our study was that the subjects of this study were patients with chronic obstructive pulmonary disease. Although home exercise programs are considered to be beneficial for the risk of falling and quality of life in older adults, in a controlled study, it was stated that exercise programs in which the physiotherapist and the older adults make decisions together increase health literacy, provide a more enjoyable exercise experience, and help patients gain greater autonomy [17]. In this paper we prefer a standardized home exercise program for all participants. In a study conducted with 171 patients with knee osteoarthritis above 60 years of age, it was found that the 3-month home exercise program was effective on the physical function and quality of life in patients who were followed by telephone [18]. In the present study, we did not investigate the effectiveness of the exercise program on the disease, since the participants with musculoskeletal system diseases that are not able to adapt to the exercise program, even if they have osteoarthritis.

In a study that continued for three months, it was found that home exercise programs followed by serial phone calls were effective in older adults who had a high risk of locomotor dysfunction [19]. Although the findings were similar to those of previous studies, the distinguishing features of the present study were its one-week intervention period and the use of daily video calls to monitor exercise compliance, prevent possible misunderstandings about exercises, and ensure continuous communication.In one study, telerehabilitation was tested on 4 patients who had COVID-19 on the Diamond Princess cruise ship and were treated in the hospital, and it was emphasized that using such inexpensive technologies in the hospital or community can be a powerful tool to overcome the social struggle related to this pandemic [20]. Differently, we included older adults without COVID-19 in our study.

In a systematic review made in 2015, it was stated that the older adults benefited from the exercise programs, but it was emphasized that the optimal exercise program is still not clear [21]. In this study, we prepared a one-week exercise program in accordance with general recommendations and older adults benefited form the exercise programs.

Depression is one of the most significant mental health disorders in the elderly. It also contributes to the morbidity of many diseases in this population. The prevalence of depression in the elderly is not lower than in other populations; in fact, in some chronic illnesses, the prevalence of depression is higher among the elderly [22]. Individuals who are physically active tend to have better mental health [13, 14]. Studies have shown that elderly individuals with high levels of physical activity exhibit lower rates of depression compared to those with low levels of physical activity [14, 23]. At the end of the exercise program of the subjects in the present study, there was an improvement in the GDS scores. This result may be due to the exercise and/or to be motivated by a professional every day.

There was no difference between before and after the program for Barthel index. We think this is because this scale detects dependency and dependency is not a situation that can improve in a short period of 1 week.

When we look at the correlation of the test parameters, we found that as age increases, performance decreases and depression increases as expected (Table 3). We thought that this is a result of sedentary lifestyle.

We found that as BMI increases, 6MWT distance decreases significantly (Table 3). It is an expected result that excess weight will reduce aerobic capacity and shorten walking distance.

There was a significant positive correlation between 6MWT, Tinetti POMA balance and Tinetti POMA walking before and after the program. This result shows that those who perform well at the beginning would have better results at the end of the program. In participants with good performance, depression would be significantly lower both before and after the program.

This research has several limitations. Although the sample size determined through power analysis was sufficient, the number of subjects may appear small. Identifying older adults who meet the current inclusion criteria proved to be quite challenging. In households without a younger individual, the ability of elderly participants to engage in video consultations via phone or internet was notably limited. Furthermore, many patients were reluctant to participate in the exercise program delivered via phone or internet. The absence of a control group represents another limitation. Additionally, the trial period was relatively short, lasting only one week, whereas exercise trials are typically conducted over several months. We were unable to conduct the 6-min walk test (6MWT) under ideal conditions, which further limited the study's findings. Close monitoring of the subjects is required for this test. We examined the story and medical records of the participants in great detail in order not to encounter an emergency. The older adults with the smallest risk assets were excluded from the study. This was one reason why the number of subjects was low. Ideal for this test is to be done on a flat surface of 30 m. Since it was not possible to meet this condition in a narrow home environment, it was tried to test in the most ideal routes possible. Test distances were lower as the number of turns was higher. However, we believe that the tests and exercises before and after the program are carried out on the same route, so it does not affect our statistical results.

## Conclusion

As a result, the older adults who are prone to sedentary life should be ensured to participate in home-based exercise programs. To follow these with distance video calling applications in physically inactive and sedentary older adults can increase the success of the exercise.

## Data Availability

The data supporting the findings of this study are available from the corresponding author upon reasonable request.
